# Circulating Elements in the Bloodstream of Growing Foals: Focus on Heavy Metals and Bone Remodeling Across Growth Stages

**DOI:** 10.1007/s12011-026-05186-3

**Published:** 2026-06-19

**Authors:** David Hulitká, Donatella Coradduzza, Andrea Sanna, Giovanni Paolo Biggio, Andrea Taras, Antonello Cannas, Ciriaco Carru, Serenella Medici, Raffaele Cherchi, Maria Grazia Cappai

**Affiliations:** 1 Chair of Animal Nutrition, Department of Veterinary Medicine, University of Sassari, via Vienna 2, Sassari, 07100 Italy; 2 Department of Biomedical Sciences, University of Sassari, Sassari, 07100 Italy; 3 SC Chimica Istituto Zooprofilattico Sperimentale della Sardegna, Via Duca degli Abruzzi, 8, Sassari, 07100 Italy; 4 Agency for Breeding and Valorization of Equines, Autonomous Region of Sardinia, piazza Lucrezia Borgia 4, Ozieri, Italy; 5 Chair of Animal Nutrition, Department of Agraria, University of Sassari, viale Italia 39, Sassari, 07100 Italy; 6 Department of Chemical, Physical, Mathematical and Natural Sciences, University of Sassari, Sassari, 07100 Italy

**Keywords:** Bone, Growth, Horse, Mineralization, Skeletal development

## Abstract

Bone tissue undergoes continuous remodelling throughout the life of the individual, and growth is the most impacting phase. In fact, tightly coordinated processes of resorption, synthesis, and mineralization of the bone matrix under systemic hormonal, biomechanical, and metabolic control occur for variable periods of time across species. The horse is a fast-growing animal and for this reason identified as precocial. It was hypothesized that blood serum levels of minerals in growing foals at different stages of accretion may vary over time. This investigation aimed to assess the dynamics of circulating mineral and trace element levels in serum from weaning to 24 months of age. A total of 40 serum samples (8 foals × 5 timepoints, T0–T4) were analyzed for total metals and metalloids by ICP-MS/MS (US EPA 6020B method). Each sample was measured in duplicate; the mean of the two replicate values was used in all subsequent statistical analyses. Cadmium (Cd) was the only trace element exhibiting statistically significant temporal variation throughout the growth period. A marked increase (*p* = 0.005) in circulating Cd was observed between T0 (6 mo.) and T1 (7 mo.), followed by a significant decline (*p* = 0.020) from T1 to T2 (12 mo.). Despite this population-level trend, no individual foal showed statistically significant intra-subject changes over time. All other essential trace elements remained stable, indicating preserved mineral homeostasis during growth from 6 to 24 months. These findings suggest that while essential minerals maintain a steady physiological profile during development, cadmium displays a distinct temporal pattern that may warrant further investigation. The data underscore the importance of monitoring heavy metals even in low-exposure environments during sensitive developmental windows.

## Introduction

 Metals naturally occurring in the environment fulfill diverse biological functions in living organisms. Essential elements such as calcium (Ca), magnesium (Mg), phosphorus (P), copper (Cu), zinc (Zn), and iron (Fe) are indispensable for proper cellular and systemic function. Among these, Fe, Zn, and Cu are particularly important trace elements involved in key metabolic activities, including enzymatic cofactoring, oxygen transport, and antioxidant defense [1]. Due to comparable physicochemical properties, divalent cations may interact in ways that affect their availability to skeletal structures [2].

In contrast, non-essential heavy metals such as lead (Pb), cadmium (Cd), and mercury (Hg) have no recognized physiological role and are considered toxic and/or carcinogenic [3, 4]. Their accumulation in tissues has been associated with adverse health effects, including disruption of bone mineral density [5].

Bone tissue undergoes continuous remodeling throughout life, involving the coordinated processes of resorption, synthesis, and mineralization of the bone matrix. The presence of metals in the organism poses a dual challenge: direct cytotoxic effects on bone cells and progressive accumulation within the bone matrix [6]. Direct toxicity primarily targets osteoblasts, leading to suppressed differentiation, reduced synthetic capacity, and impaired mineralization. The influence on osteoclasts varies by element; certain metals may enhance or inhibit TRAP activity and disrupt precursor maturation [7]. Such alterations may disturb the balance of bone remodeling, contributing to skeletal disorders. The capacity of trace metals to accumulate within the extracellular matrix prolongs their biological half-life, which is particularly relevant under low chronic exposure [8].

The cellular architecture of bone includes osteoblasts and osteocytes (formation and maintenance) and osteoclasts (resorption and remodeling). The organic matrix, or osteoid, consists primarily of collagen fibrils into which mineral salts are deposited during mineralization. Longitudinal growth occurs within the epiphyseal growth plates, where chondrocytes are progressively replaced by mineralized primary bone through chondrocyte apoptosis, matrix calcification, and vascular invasion [9–11].

Thoroughbred foals are born at approximately 10% of adult body mass; by six months they reach ~ 45–55%, and by two years approximately 96% of adult weight. Between weaning (5–6 months) and two years, skeletal accretion is particularly rapid [12, 13], creating a developmental window of potential susceptibility to disruptions in mineral metabolism, including exposure to non-essential heavy metals.

Cd and Pb, in particular, interfere with osteoblast differentiation, inhibit collagen and alkaline phosphatase synthesis, generate oxidative stress, and may alter osteoclast activity. Competing interactions between essential and toxic metals—such as the Cd/Zn competition for transporters or Pb substitution for Ca in hydroxyapatite—further underscore the rationale for monitoring these elements during critical developmental periods. Very little is known about heavy metal dynamics in young horses during growth and whether such shifts can be detected systemically in blood.

It was hypothesized that circulating levels of minerals in growing foals at different stages of accretion may vary over time. The objective of the present study was to evaluate the dynamics of circulating mineral and trace element concentrations in serum and to assess their relationship with biological parameters in foals during the growth period between 6 and 24 months of age.

## Materials and Methods

### Animal Welfare and Ethical Statement

All procedures conducted in this study complied with the European Union Directive 2010/63/EU on the protection of animals used for scientific purposes. Animal handling and experimental procedures, including blood sampling, biometric evaluation, and radiographic assessments, were part of routine clinical practice and were performed exclusively by licensed veterinary professionals and trained technicians. The study protocol adhered to the ethical standards outlined by the International Committee of Medical Journal Editors (ICMJE, 2006) for the conduct and publication of biomedical research. The study was strictly observational and involved a total of 40 serum samples (8 foals × 5 timepoints), collected during routine management procedures for sanitary reasons, with no experimental treatment imposed.

### Animals and Experimental Design

Eight healthy Anglo-Arab foals (initial body mass: 191 ± 15.7 kg; age at baseline: 25–26 weeks) born and reared at the experimental facility of the Agency for Horse Breeding and Valorization of the Autonomous Region of Sardinia were enrolled. Inclusion criteria of animals in the trial took into account the following factors: (1) same breed; (2) same birth period (all foals were born within a week in the month of March); and (3) mares were reared under the same breeding conditions during pregnancy and throughout lactation until foal’s weaning, as a prerequisite to include the foal in the investigation.

### Management and Feeding Protocol

Until T0 phase, the totality of suckling foals spent the first 6 months of life with the mare. Foals with mare grazed together on the same pasture (31 hectares), from April to October. Foals’ diet was based essentially on mare’s milk and fresh fodder in Spring, whereas in Summer, home-grown hay (oat, and clover based hay) and concentrate were administered to lactating mares with foals. Throughout the suckling period, all foals were kept on pasture during daytime and sheltered in individual boxes with the mare at night.

In the T1 phase, foals (29–30 weeks old) were weaned and housed in individual boxes, where they were fed with a conventional dietary treatment for weanlings, *ad libitum*. The daily feeding practices consisted of home-grown hay and concentrate. Concentrates were weighed and administered manually through a feeder suspended in each box.

In the T2 phase, foals were sent to pasture and were supplemented with concentrate feed when stabled, offered to assure a daily leftover, weighing not less than 100 g. This feeding regime was maintained until foals were 1 year old.

Across the three phases of the observational period, animals were clinically assessed for overall health conditions and nutritional-metabolic status, supported by complete hematological and biochemical profiles. Feeding practices ensured consistent nutrient availability and minimized variability in mineral intake. This standardized feeding regime ensured minimized variability in mineral and heavy-metal intake among animals, over time. Table 1 reports hay and compound feed composition and administration practices.

**Table 1:** Feeding plan and feedstuffs used in the different stages of the investigation

Item 2	Age ^1^			
	6 mo.	12 mo.	18 mo.	24 mo.
Ingredient (kg/d per horse, as fed)				
Hay	4.00	5.00	7.00	8.00
Compound feed	3.00	4.00	4.00	3.00
Pasture	*—*	+	+	+
Chemical composition of compound feed				
(g/kg feed)				
DM	881	883		
NDF	290	453		
CP	213	345		
Ash	66.2	138		
Ether extract	70.1	26.4		
DE (Mcal/kg DM) ^3^	3.64	3.09		
Chemical composition of hay				
(g/kg feed)				
DM	847			
NDF	585			
CP	129			
Ash	135			
Ether extract	21.6			
DE (Mcal/kg DM) ^3^	2.44			

^1^ Age: 6 mo., foal aged six months at weaning; 12 mo., foal at one year; 18 mo., foal aged 18 months; 24 mo, foals aged 24 months

^2^ Item: DM, dry matter; NDF, neutral detergent fiber; CP, crude protein

^3^ DE = digestible energy per kg of dry matter in feed expressed as mega calories. Energy density for concentrate and forage was predicted by the formula of NRC, 2007

### Clinical Monitoring and Blood Sampling

**Fig. 1:**
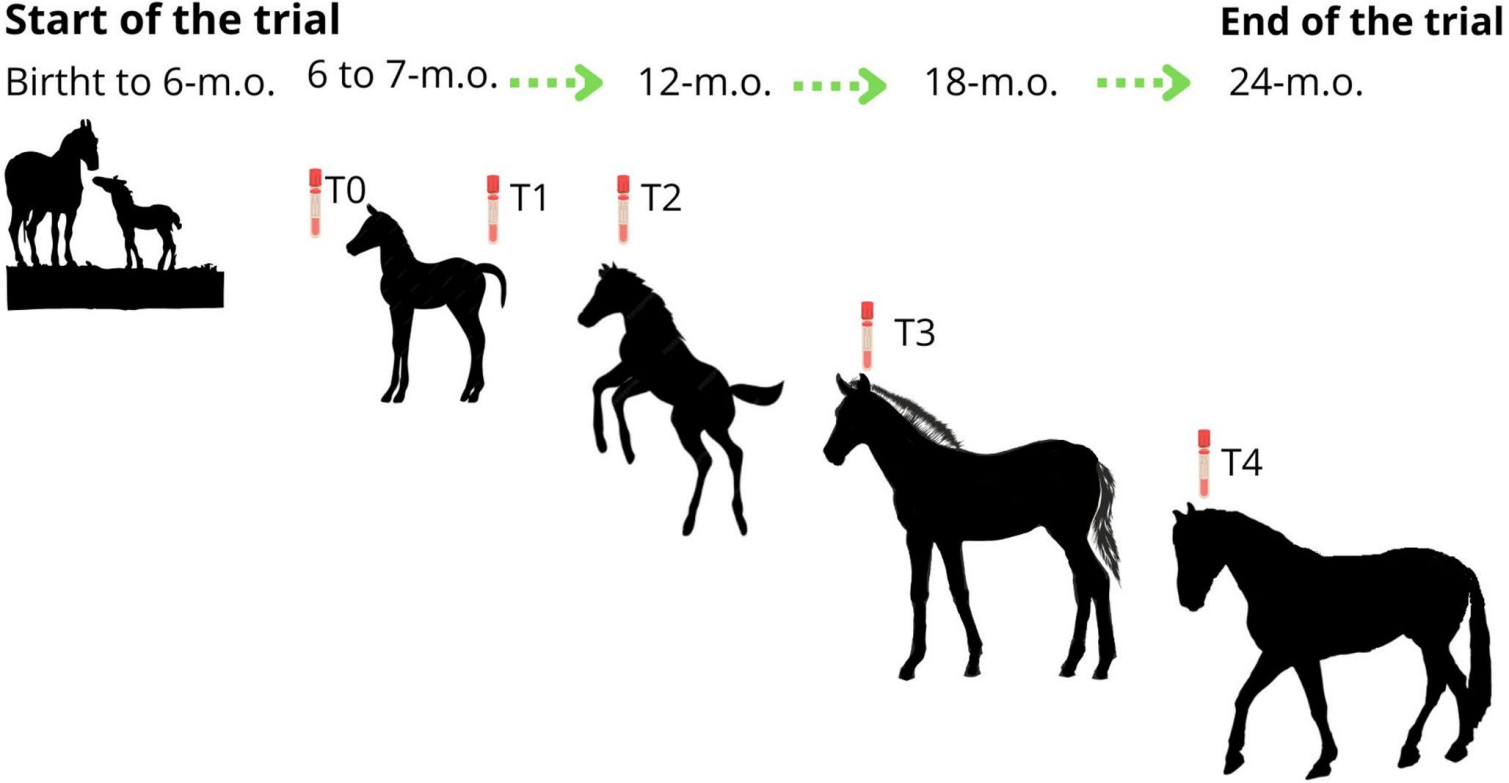
Screening plan of growth stages across timepoints of individual checks. A total of 8 horses were involved and 40 samples were collected

Animals underwent regular clinical evaluations, including hematological and biochemical profiling, across all trial phases. Blood was collected as serum at each timepoint via jugular venipuncture. To account for potential circadian variation in biochemical parameters [14], each foal was sampled twice daily: at 08:00 (2 h post-hay) and at 19:00. For each foal at each timepoint, the two daily serum samples were analyzed as independent replicates; the mean of the duplicate measurements was used as the representative value for that foal at that timepoint in all subsequent statistical analyses. Figure 1 presents the timetable of experimental phases and sampling time, throughout the trial.

### Heavy Metals Serum Analysis Using ICP-MS

Total metals and metalloids in serum (aluminium, antimony, arsenic, barium, beryllium, cadmium, chromium, cobalt, copper, iron, lead, lithium, manganese, mercury, nickel, molybdenum, selenium, silver, thallium, tin, vanadium and zinc) were determined by inductively coupled plasma mass spectrometry (ICP-MS) in accordance with the US EPA 6020B method26. Biological fluids were analysed directly after dilution of 0.5 mL of sample in 5 mL with 2% nitric acid (J.T. Baker, Phillipsburg, NJ, USA) solution. The analysis was performed with an inductively coupled plasma mass spectrometer ICP-MS/MS (Agilent 8800 QQQ, Santa Clara, CA, USA) equipped with a collision cell and two quadrupole mass analyzers. In comparison to a single quadrupole ICP-MS system, the triple quadrupole system significantly increases the accuracy of mass separation. To compensate for the matrix effect and signal drift, a solution of internal standards was used. The calibration curve was verified at the start of each analytical batch using the initial calibration verification (ICV) with a different lot standard, while the instrumental sensitivity was verified using the continuous calibration verification (CCV) at or near midrange. The LOQs testing was 0.001 ng/mL for all elements analysed. All samples were run in duplicate, and the mean value was used for statistical analysis. The quality control of the data was verified and controlled using Certified Reference Materials ClinChek^®^ Urine Control, and ClinChek^®^ Serum Control for Trace Elements, (RECIPE Chemicals, München). The laboratory was intercalibrated through successful participation in internationally organized proficiency tests (OELM). The method is accredited according to UNI EN ISO 17,025/2017.

### Statistical Analysis

A total of 40 paired serum measurements (8 foals × 5 timepoints, T0–T4) were included in the statistical analysis. Data quality was assessed through completeness checks, outlier inspection, and evaluation of distributional properties. Normality was tested using the Shapiro–Wilk test and visual diagnostics (Q–Q plots, histograms). When non-normality was detected, log-transformation was applied; otherwise, non-parametric analyses were used. Temporal changes were evaluated using one-way repeated-measures ANOVA, with foal ID included as the within-subject factor. When model assumptions were violated, the Friedman test was used. Post-hoc pairwise comparisons between consecutive phases (T0–T1, T1–T2, T2–T3, T3–T4) employed Tukey’s HSD correction for parametric analyses or Bonferroni-adjusted Wilcoxon tests for non-parametric ones. Multivariate relationships were explored using Principal Component Analysis (PCA) performed on mean-centered but non-scaled variables, as all trace elements were expressed in the same unit (ng/mL). Pearson correlation matrices and heatmaps were generated to visualize inter-element covariation patterns. Statistical significance was set at *p* < 0.05, with 0.05 ≤ *p* < 0.10 considered a trend. All analyses were performed using MedCalc (v.22.0).

## Results

A total of eight foals were monitored across five predefined growth phases (T0–T4), yielding 40 serum samples for analysis. Concentrations of 20 trace elements and heavy metals were quantified using ICP-MS. After data cleaning, verification of completeness, and restructuring of the dataset, all analytical variables were retained for statistical evaluation.

### Descriptive Analysis Across Timepoints

Descriptive statistics (mean, median, SD) were generated for each trace element at T0–T4. At the population level, the majority of both essential and non-essential elements exhibited relatively stable circulating serum concentrations across growth phases. Nonetheless, several analytes displayed greater dispersion, particularly during the transition from pre-weaning to post-weaning (T0–T1) and throughout the early post-weaning period (T1–T2). Among the 20 quantified elements, a subset showed the largest interquartile variation across time. Despite these observations, only one element—cadmium (Cd)—demonstrated statistically significant temporal modulation in the overall repeated-measures analysis, while all others remained stable across phases (Table 2).

**Table 2:** Plasmatic circulating values of trace elements in samples across developmental phases (T0–T4)

Timepoint sample Concentration (ng/ml) Elements	T0 Mean ± SD Median		T1 Mean ± SD Median		T2 Mean ± SD Median		T3 Mean ± SD Median		T4 Mean ± SD Median	
Lithium	6.07 ± 1.85	6.05	5.54 ± 1.95	5.68	6.92 ± 2.16	7.15	6.23 ± 1.72	5.86	5.60 ± 1.73	5.55
Beryllium	0.00 ± 0.00	0.00	0.00 ± 0.00	0.00	0.00 ± 0.00	0.00	0.00 ± 0.00	0.00	0.00 ± 0.00	0.00
Manganese	3.14 ± 0.37	3.18	3.15 ± 0.48	3.16	2.77 ± 0.34	2.82	2.98 ± 0.25	3.04	2.89 ± 0.30	2.92
Cobalt	1.57 ± 0.73	1.36	1.58 ± 0.75	1.32	1.54 ± 0.69	1.31	1.54 ± 0.61	1.39	1.53 ± 0.61	1.34
Vanadium	0.09 ± 0.06	0.07	0.07 ± 0.02	0.07	0.07 ± 0.02	0.07	0.08 ± 0.02	0.07	0.07 ± 0.01	0.07
Chromum	1.43 ± 0.54	1.39	1.05 ± 0.78	1.07	1.28 ± 0.81	1.11	1.21 ± 0.78	1.10	1.49 ± 0.72	1.33
Iron	1857 ± 332	1899	2430 ± 557	2266	1996 ± 354	1882	1985 ± 207	1968	2102 ± 769	1937
Nickel	1.25 ± 1.14	1.26	1.44 ± 1.03	1.95	0.85 ± 0.88	0.45	1.32 ± 1.06	1.79	1.26 ± 1.25	1.37
Copper	756 ± 90.4	767	768 ± 94.7	777	751 ± 85.9	753	738 ± 70.8	726	751 ± 71.7	755
Zinc	492 ± 130	471	448 ± 141	414	440 ± 104	424	441 ± 140	430	418 ± 89.5	422
Selenium	101 ± 27.0	93.9	107 ± 30.0	98.59	100 ± 25.5	97.9	103 ± 17.3	99.7	101 ± 22.9	97.5
Arsenic	0.95 ± 0.19	0.92	0.95 ± 0.13	1.02	0.92 ± 0.24	0.94	1.07 ± 0.17	1.14	1.01 ± 0.13	1.01
Molybdenum	7.85 ± 5.15	5.54	6.83 ± 4.71	5.21	8.95 ± 6.88	5.74	7.11 ± 4.52	5.86	7.62 ± 4.96	5.74
Cadmium	0.09 ± 0.02	0.09	0.12 ± 0.01	0.11	0.10 ± 0.01	0.10	0.10 ± 0.01	0.10	0.11 ± 0.02	0.11
Tin	0.00 ± 0.00	0.00	0.00 ± 0.00	0.00	0.00 ± 0.00	0.00	0.00 ± 0.00	0.00	0.00 ± 0.00	0.00
Antimony	14.8 ± 1.78	14.7	15.7 ± 1.56	15.3	14.4 ± 1.19	14.3	15.1 ± 1.49	15.1	15.3 ± 3.62	14.4
Barium	7.98 ± 3.15	6.87	7.02 ± 2.21	6.56	6.79 ± 2.07	6.45	7.01 ± 1.46	6.81	5.87 ± 1.51	5.80
Mercurium	0.11 ± 0.06	0.10	0.09 ± 0.03	0.11	0.09 ± 0.03	0.09	0.11 ± 0.05	0.10	0.09 ± 0.04	0.08
Thallium	0.02 ± 0.02	0.02	0.02 ± 0.02	0.02	0.02 ± 0.02	0.03	0.02 ± 0.02	0.01	0.02 ± 0.01	0.02
Lead	2.10 ± 0.16	2.06	2.41 ± 0.51	2.23	1.95 ± 0.12	1.98	2.13 ± 0.16	2.06	2.22 ± 0.44	2.08

Values represent serum concentrations expressed in ng/mL. For each analyte, descriptive statistics were calculated across the five developmental phases (T0–T4). “—” indicates values below the limit of quantification (LOQ). Cadmium values show the characteristic T0 to T1 increase and T1to T2 decline described in the text

### Temporal Trends and ANOVA measures

RM-ANOVA identified cadmium (Cd) as the only analyte with a statistically significant temporal effect (*F* = 3.58, *p* = 0.015). For all remaining elements, RM-ANOVA revealed no significant differences across timepoints (Table 2). Post-hoc testing confirmed Cd as the sole element maintaining statistical significance after multiple-comparison correction.

### Post-hoc Pairwise Comparisons for Cadmium

Post-hoc pairwise comparisons confirmed that cadmium exhibited its most pronounced temporal fluctuations during the early stages of growth. A significant increase in Cd concentrations was observed from T0 to T1 (*p* = 0.005), indicating a marked increase in circulating Cd immediately following weaning. This was followed by a significant decline between T1 and T2 (*p* = 0.020).The comparison between T0 and T3 showed only a trend toward significance (*p* = 0.085), while T2 to T3 did not reveal meaningful variation (*p* = 0.542). The T3–T4 comparison showed no significant difference (*p* = 0.591).Taken together, these results describe a biphasic temporal pattern, characterized by an increase in circulating Cd between T0 and T1 followed by a decline between T1 and T2 and subsequent stabilization.

### ANOVA and post-hoc Overview for All Elements

**Fig. 2:**
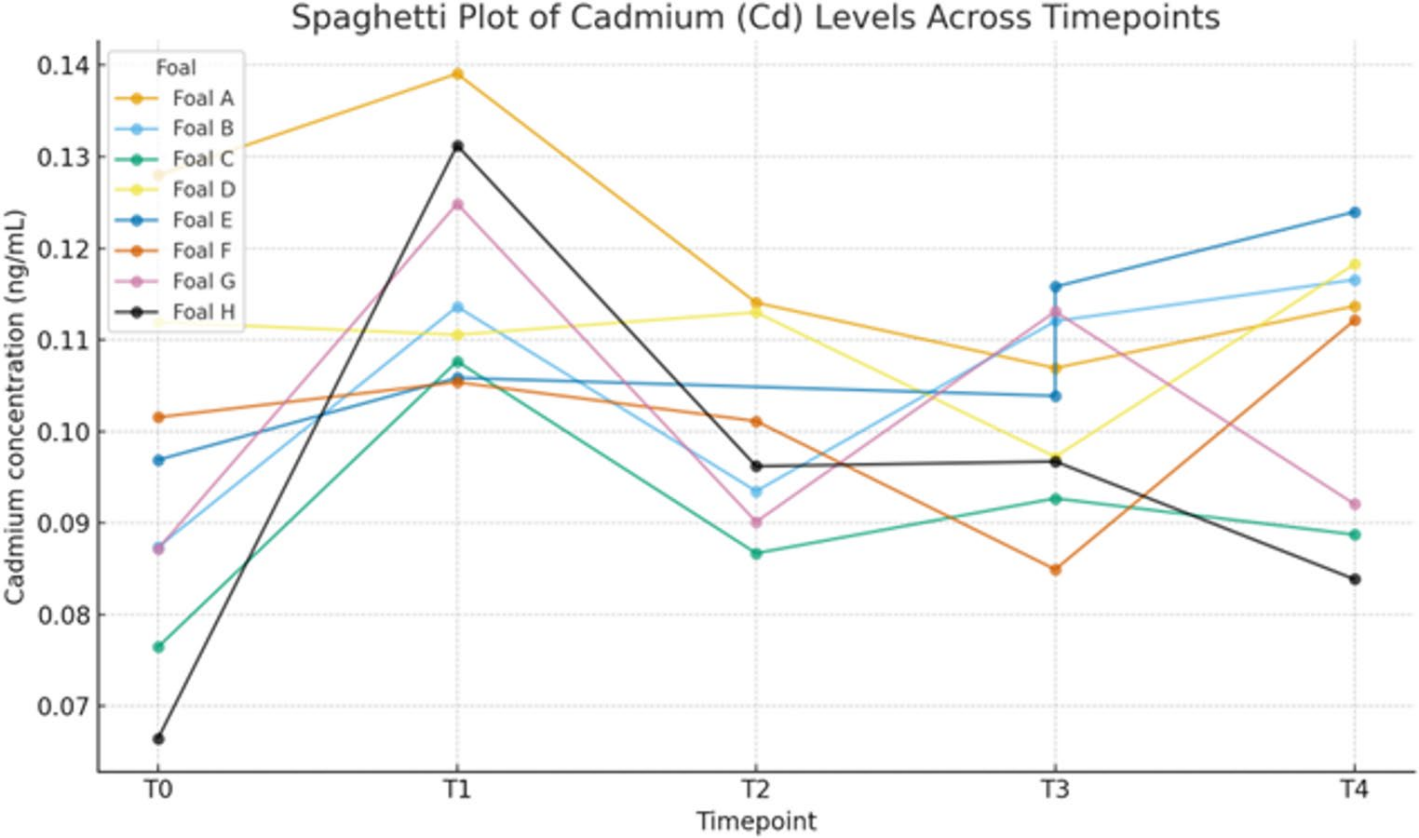
Longitudinal trajectories of cadmium (Cd) concentrations across growth phases in individual foals. Spaghetti plot illustrating serum cadmium concentrations measured in each foal (**A**–**H**) across the five sampling timepoints (T0–T4). Each line represents the intra-subject temporal trajectory, while points correspond to individual measurements obtained through ICP-MS. All foals exhibit a similar temporal pattern, characterized by a pronounced increase at T1 (post-weaning) followed by a decline toward T2, supporting the population-level trend identified through ANOVA. Despite the common group trajectory, intra-individual variability remained insufficient to achieve statistical significance within each subject. This visualization highlights the collective, rather than individual, nature of cadmium modulation during the growth period

**Fig. 3:**
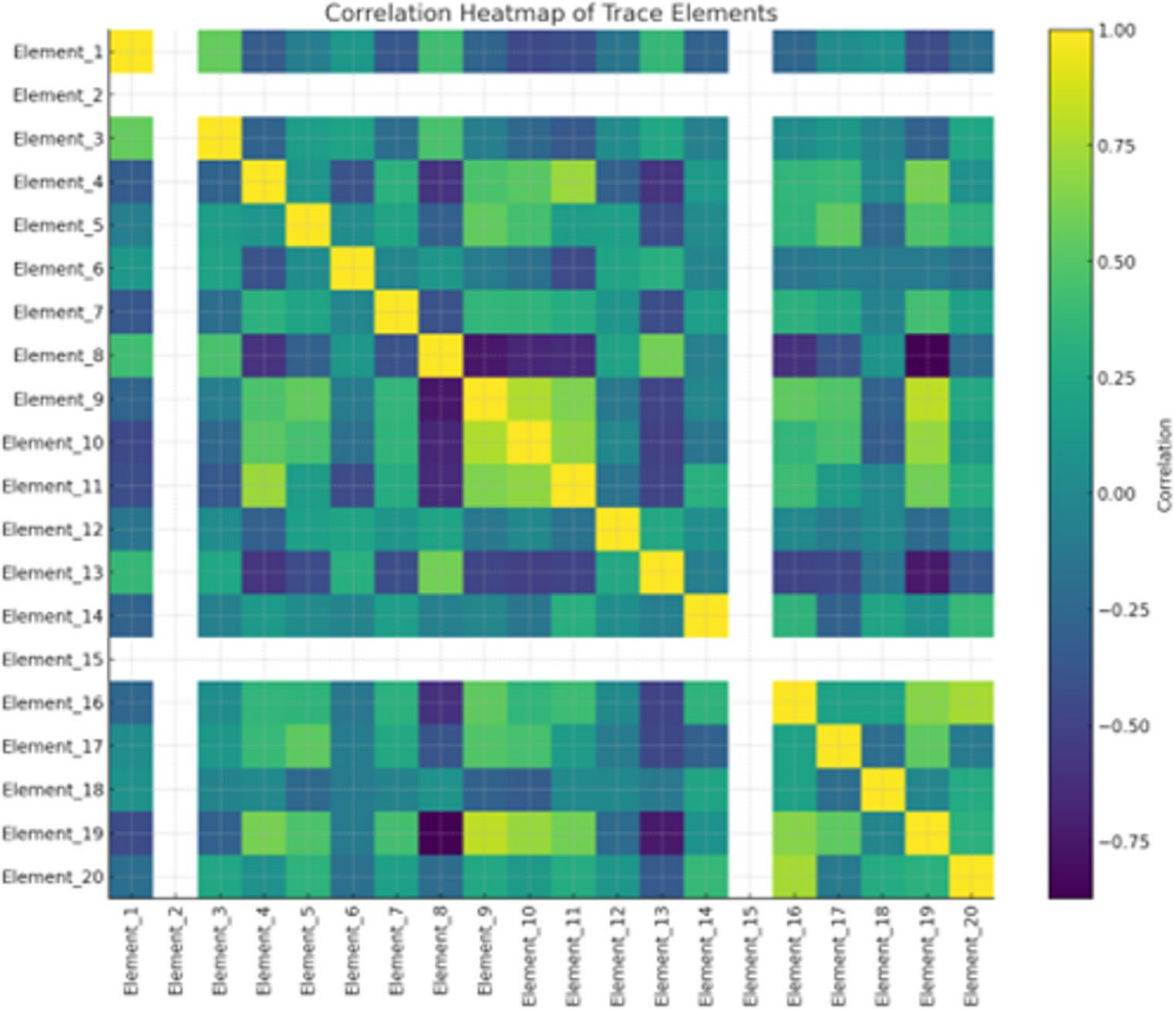
Correlation heatmap of serum trace elements in growing foals. The heatmap illustrates pairwise correlation coefficients among the 20 quantified serum trace elements and heavy metals measured across all growth phases (T0–T3). Colors range from dark blue (strong negative correlation) to bright yellow (strong positive correlation), with green tones indicating weak or no correlation. Each cell represents the Pearson correlation between two elements, computed using all available samples. Distinct clusters of positively correlated elements are visible, suggesting shared physiological regulation or common environmental sources, whereas isolated patterns—such as those observed for cadmium—indicate independent temporal dynamics. This visualization provides an overview of elemental interrelationships throughout the growth period

A comprehensive summary of the ANOVA models and post-hoc comparisons for all quantified trace elements is presented in Table 3. The analysis included a total of 40 serum samples (8 foals × 5 timepoints, T0–T4). Cadmium (Cd) was the only analyte exhibiting statistically significant temporal variation across developmental stages. No statistically significant differences across timepoints were observed for the remaining elements. To complement the univariate statistical analysis and visually assess intra-subject dynamics, a spaghetti plot of individual Cd trajectories is presented in Fig. 2. Individual trajectories showed an increase at T1 followed by a decrease at T2 (Fig. 3).

**Table 3:** One-way repeated-measures ANOVA across developmental phases (T0–T4)

Element	F-value	*p*-value	Significance
Lithium	n.s.	0.620	Not significant
Beryllium	—	—	Not significant (all values below LOQ)
Manganese	n.s.	0.198	Not significant
Cobalt	n.s.	0.999	Not significant
Vanadium	n.s.	0.809	Not significant
Chromium	n.s.	0.767	Not significant
Iron	n.s.	0.191	Not significant
Nickel	n.s.	0.870	Not significant
Copper	n.s.	0.964	Not significant
Zinc	n.s.	0.819	Not significant
Selenium	n.s.	0.982	Not significant
Arsenic	n.s.	0.428	Not significant
Molybdenum	n.s.	0.945	Not significant
Cadmium	**3.58**	**0.015**	**Significant temporal modulation**
Tin	—	—	Not significant (all values below LOQ)
Antimony	n.s.	0.784	Not significant
Barium	n.s.	0.437	Not significant
Mercury	n.s.	0.771	Not significant
Thallium	n.s.	0.989	Not significant
Lead	n.s.	0.104	Not significant (trend only)

Values represent results of one-way repeated-measures ANOVA testing for temporal differences across the five developmental phases (T0–T4). “—” indicates elements consistently below the limit of quantification (LOQ). Cadmium (Cd) was the only analyte exhibiting a statistically significant effect of time (*p* < 0.05), displaying the characteristic T0→T1 increase followed by a decline at T2. All remaining trace elements remained stable across the growth period

### Individual-Level Temporal Variation

No trace element demonstrated statistically significant intra-subject fluctuations across any foal (A–H) at adjacent timepoints (T0–T1, T1–T2, T2–T3; Table 4). Results for each individual are reported in Table 4.

**Table 4:** ANOVA and post-hoc comparisons for trace elements at each single timepoint sampling

Timepoint sample Elements	T0 – T4	T0 -T1	T1 -T2	T2 -T3	T3 – T4
Lithium	0.620	0.585	0.218	0.502	0.462
Beryllium	< LOD	< LOD	< LOD	< LOD	< LOD
Manganese	0.198	0.968	0.099	0.205	0.541
Cobalt	1.000	0.975	0.896	0.989	0.968
Vanadium	0.809	0.518	0.921	0.675	0.487
Chromum	0.7669	0.287	0.600	0.879	0.455
Iron	0.191	**0.0287**	0.092	0.944	0.686
Nickel	0.870	0.731	0.250	0.350	0.920
Copper	0.964	0.795	0.733	0.733	0.708
Zinc	0.819	0.526	0.908	0.984	0.682
Selenium	0.982	0.699	0.623	0.800	0.875
Arsenic	0.428	0.932	0.772	0.182	0.422
Molybdenum	0.945	0.685	0.506	0.553	0.829
Cadmium	**0.035**	**0.017**	**0.011**	0.542	0.591
Tin	< LOD	< LOD	< LOD	< LOD	< LOD
Antimony	0.784	0.290	0.081	0.317	0.876
Barium	0.437	0.495	0.835	0.811	0.137
Mercurium	0.771	0.628	0.820	0.353	0.275
Thallium	0.989	0.897	0.696	0.957	0.746
Lead	0.104	0.134	**0.040**	**0.023**	0.605

Bold values highlight the statistic significance

### Principal Component Analysis

**Fig. 4:**
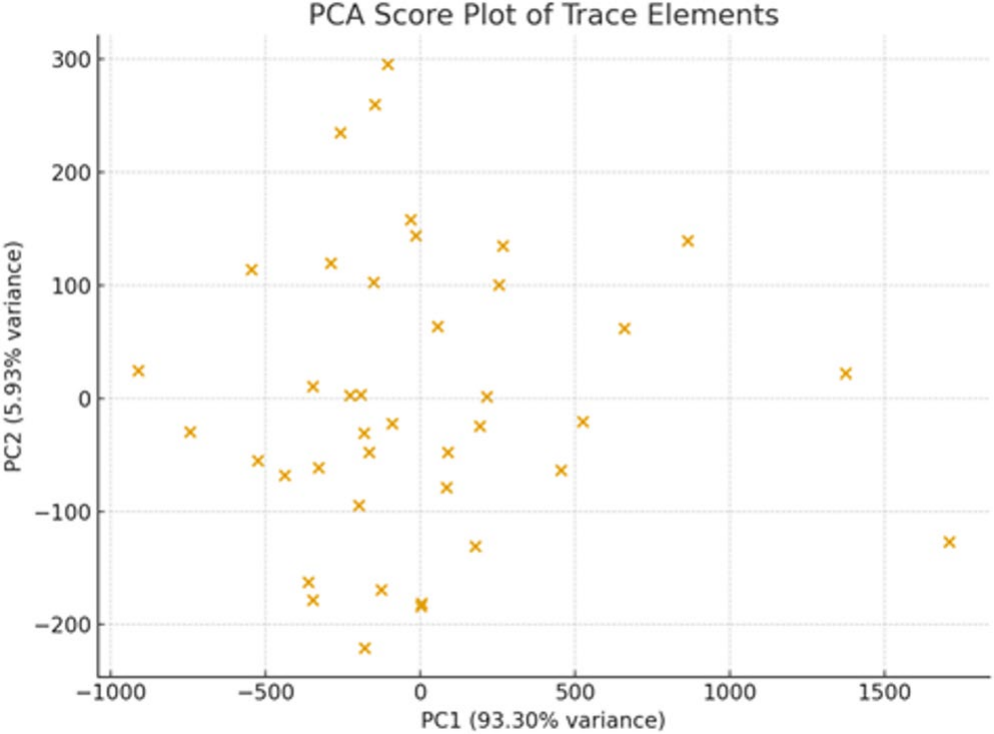
PCA score plot for serum trace elements. Score plot representing individual sample projections onto PC1 and PC2. Samples corresponding to different timepoints overlap extensively, indicating the absence of temporal clustering and highlighting the stability of the multielement profile during growth

**Fig. 5:**
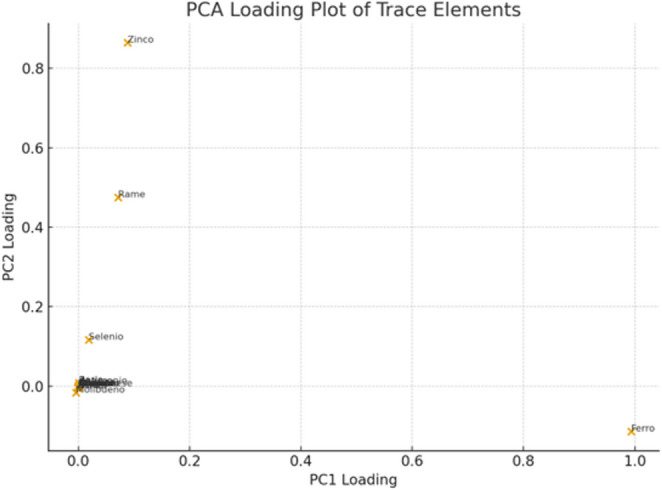
PCA loading plot showing contributions of each element to the PCA model. Iron dominates PC1, while zinc and copper contribute primarily to PC2. The clustering of remaining elements near the origin reflects minimal variance across the dataset

A Principal Component Analysis (PCA) was performed on 40 serum samples. PC1 explained 93.30% of total variance; PC2 explained 5.93%; together they accounted for 99.23%. The PCA score plot (Fig. 4) shows no separation or clustering by developmental phase. The loading plot (Fig. 5) identifies Fe as the strongest contributor to PC1; Zn and Cu loaded predominantly on PC2. Remaining analytes clustered near the origin.

## Discussion

The present study provides the first longitudinal characterization of circulating trace elements and heavy metals in serum of growing foals from weaning to late juvenile development (T0–T4). Among the 20 quantified analytes, cadmium (Cd) emerged as the sole element exhibiting statistically significant temporal modulation. The observed biphasic pattern—an abrupt rise at T1 (post-weaning) followed by partial normalization at T2—represents a statistically significant but transient variation occurring during the early post-weaning period. All other essential and non-essential elements remained stable across growth, highlighting the robustness of mineral homeostasis in young horses even during phases of rapid skeletal development.

### Cadmium and Skeletal Development: Biological Plausibility

The significant increase in serum Cd from T0 to T1 and subsequent decline at T2 represents the central finding of this study. While the mechanistic basis of this pattern cannot be established from the current observational dataset, several physiological processes are compatible with the observed temporal dynamics.

Extensive toxicological literature documents that Cd exposure can disrupt Ca and P homeostasis, impair bone mineralization, and interfere with vitamin D metabolism, as documented at toxic exposure levels [14–17]. Whether these pathways are activated at the low Cd concentrations observed in this cohort remains unknown and cannot be established from the current observational data.

The transient rise at T1 may be compatible with several converging post-weaning changes. Among these, one temporally coherent possibility is the dietary transition from mare’s milk and fresh forage to mineral-enriched compound concentrates specifically formulated for weanlings. Cadmium is classified as an “undesirable substance” under EU Directive 2002/32/EC – not a prohibited contaminant – with a maximum tolerable level of 1 mg/kg (88% DM) for compound feed. This regulatory definition acknowledges that Cd is an inevitable co-contaminant in commercial feed matrices, particularly those containing mineral phosphate supplements and phosphate fertilizer-derived raw materials, which are known to carry Cd as a natural impurity in phosphate rock [18]. The temporal coincidence between T1 and the introduction of weanling concentrate is consistent across all eight subjects, supporting a plausible, if unconfirmed, dietary exposure route. Importantly, even if dietary Cd measurements had been performed, a simple linear dose-response relationship between feed Cd content and serum Cd concentration would not have been scientifically defensible, given the inherently non-linear kinetics of Cd absorption, redistribution, and excretion, modulated by age, gastrointestinal maturation, metallothionein induction, and renal handling. Additional hypotheses include: (ii) increased soil and forage ingestion upon independent grazing; (iii) possible redistribution of Cd accumulated during suckling; or (iv) transient shifts in renal clearance during metabolic adaptation. The subsequent decline at T2 may reflect progressive re-establishment of homeostatic equilibrium. These interpretations are speculative and cannot be confirmed in the absence of environmental sampling and pharmacokinetic data. Within the limits of this study, no causal attribution can be made.

### Competition with Essential Trace Elements

Divalent heavy metals interact with essential trace element pathways: Cd and Zn share molecular carriers, while Cd and Cu compete for intestinal absorption [14–17]. Although no essential mineral showed statistically significant temporal variation in this cohort, these competitive interactions are noted as a literature-contextualized framework for future investigation, rather than as a conclusion supported by the present data.

### Multielement Stability and PCA Findings

The stability of 19 out of 20 elements across all timepoints represents a consistent observation within the limits of this study. Essential elements including Fe, Cu, Zn, Se, and Mn remained tightly regulated despite major physiological changes during growth. PCA confirmed the absence of coordinated temporal shifts, with timepoints extensively overlapping. Fe showed the strongest loading on PC1, consistent with its biological prominence in erythropoiesis, oxygen transport, and skeletal muscle expansion during growth—processes that dominate systemic iron demand in this developmental window. Zn and Cu loaded primarily on PC2, reflecting their partial regulatory independence from the broader variance structure; this pattern is compatible with their distinct homeostatic control via metallothionein (Zn) and ceruloplasmin (Cu), which buffer circulating concentrations against dietary and metabolic fluctuations through mechanisms that operate largely independently of iron. The remaining analytes clustered near the origin. These findings indicate that the overall multielement profile remained stable throughout development.

### Contextualizing the Detected Cd Levels

The absolute Cd concentrations were low relative to values reported in equids from industrially impacted regions, consistent with the geographic isolation of the breeding facility and controlled forage sources. The concentrate and hay provided were below the Cd threshold established by Directive 2002/32/EC (1 mg/kg, 88% DM) [18]. The multielement stability observed further suggests that dietary sources did not exert significant toxicological pressure during the study period. Despite the low absolute exposure, the temporal pattern of Cd was distinct and may warrant monitoring in future cohort studies.

## Limitations

Several methodological limitations should be considered when interpreting the findings of the present study. First, the relatively small sample size (*n* = 8) inherently limits the statistical power of the analysis, particularly with regard to detecting intra-individual variation across timepoints. As a consequence, the absence of statistically significant within-subject changes—especially for cadmium—should be interpreted with caution, as subtle biological effects may have remained undetected. Moreover, the fact that cadmium emerged as the only element showing significant temporal variation may partly reflect this limited statistical power rather than a true biological singularity. A further limitation concerns the lack of direct biomarkers of bone metabolism. No indices of bone formation or resorption, such as osteocalcin, bone-specific alkaline phosphatase, or collagen degradation markers, were assessed. Therefore, any inference regarding the potential relationship between cadmium dynamics and bone remodeling processes remains indirect and should be interpreted cautiously. Similarly, the absence of vitamin D metabolite measurements precludes any evaluation of endocrine mechanisms potentially linking cadmium exposure to calcium–phosphorus homeostasis. In addition, renal tubular biomarkers indicative of early cadmium-induced nephrotoxicity were not included in the study. Although routine biochemical parameters did not reveal overt renal dysfunction, the possibility of subclinical tubular effects cannot be excluded. The lack of environmental measurements represents another important limitation. Feed, water, soil, and pasture were not analyzed for cadmium content, preventing accurate estimation of exposure sources and intake levels. It should be noted, however, that the identification of this post-weaning cadmium pattern was entirely unexpected and emerged from an hypothesis-free observational design; environmental sampling had therefore no scientific basis to be planned prospectively. Furthermore, even if dietary cadmium measurements had been performed, a direct linear attribution from dietary intake to serum concentration would not have been scientifically defensible, given the inherently non-linear kinetics of cadmium absorption, redistribution, and excretion, which are modulated by age, gastrointestinal maturation, metallothionein induction, and renal handling. Consequently, any interpretation regarding the origin of the observed post-weaning increase in circulating cadmium remains speculative. Finally, the observational nature of the study does not allow causal relationships to be established. The temporal association observed for cadmium should therefore be interpreted as descriptive of a population-level trend rather than indicative of a direct biological effect. Future investigations integrating larger cohorts, environmental monitoring, bone turnover biomarkers, vitamin D metabolites, and renal tubular markers will be essential to clarify the mechanistic and clinical relevance of these findings.

## Conclusion

The present longitudinal analysis demonstrates that clinically healthy growing foals maintain stable circulating serum concentrations of all evaluated trace elements except cadmium, which displays a short-lived but statistically significant biphasic fluctuation in the immediate post-weaning period. This pattern, consistent across all eight subjects at the population level, has not been previously described in the equine species. Among the plausible explanations for the post-weaning cadmium peak, the dietary transition to mineral-enriched weanling compound concentrates, which are known to carry cadmium as an unavoidable trace contaminant under EU Directive 2002/32/EC, represents a temporally coherent, though unconfirmed, possibility; no causal attribution can be made from the current observational dataset, and the mechanistic basis and functional significance of this finding remain to be established. The detected Cd levels were substantially below those associated with clinical toxicity in equids, and all other essential and non-essential trace elements remained in stable homeostasis throughout the growth period studied. These findings should be considered hypothesis-generating for future studies essential to characterize the biological relevance of cadmium kinetic and its potential implications for musculoskeletal development in the growing horse.

## Data Availability

Data of this investigation are reported in full in the manuscript.

## Declarations

### AI use

Authors declare that AI tool was used for the cross check of data analysis.
